# Supplementary material for: Cx43-mediated hyphal folding counteracts phagosome integrity loss during fungal infection
Source: Microbiol Spectr. 2023 Sep 21;11(5):e01238-23. doi: 10.1128/spectrum.01238-23 (PMC10581180; doi:10.1128/spectrum.01238-23)
Supplement: Supplemental Figures — Fig. S1 to S7 [file spectrum.01238-23-s0001.pdf]

Supplementary Data

Cx43-mediated hyphal folding counteracts phagosome integrity loss during fungal infection

Beatriz Cristovao[1,2,†], Neuza Domingues[1,2†], Lisa Rodrigues[2,3], Mónica Abreu[1,2] ,Teresa Gonçalves[3,4], Henrique Girao[1,2*]

[1] Faculty of Medicine, Coimbra Institute for Clinical and Biomedical Research, Center for Innovative Biomedicine and Biotechnology, University of Coimbra, Coimbra, Portugal

[2] University of Coimbra, Academic and Clinical Center of Coimbra, Coimbra, Portugal

[3] CNC-UC, Center for Neurosciences and Cell Biology, Coimbra, Portugal

[4]FMUC – Faculty of Medicine, University of Coimbra, Coimbra Portugal

[*] Corresponding author: hmgirao@fmed.uc.pt

[†] These authors contributed equally to the work

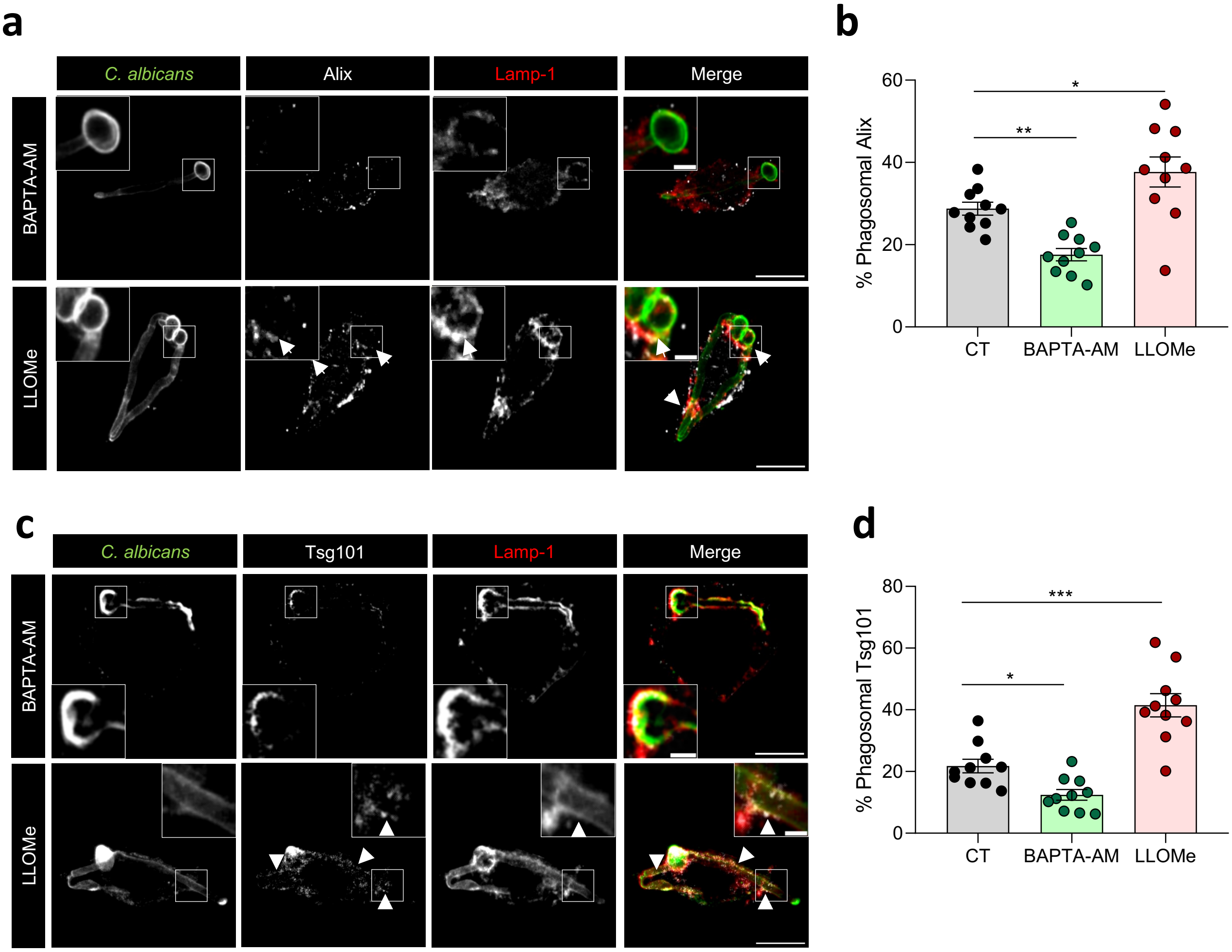

FIG S1 Recruitment of ESCRT machinery proteins to the hyphae-containing phagolysosomes. (a and d) Representative confocal images of RAW cells infected with *C. albicans* and treated with BAPTA-AM and LLOMe, fixed 180 min post-infection, and immunostained for *C. albicans*, ALG-2-interacting protein X (Alix) (a), Tumor susceptibility gene 101 protein (Tsg101) (d), and Lamp-1. Scale bar, 10 μm and 2 μm in the insets images. White arrows indicate sites of Alix and Tsg101 accumulation. (c and d) Quantification of the percentage of phagosomal Alix (c) and Tsg101 (d) in control condition and after BAPTA-AM and LLOMe treatment quantified as (phagosomal protein of interest/total protein of interest)*100 per cell. Results represent the mean ± SEM from 10 cells. p-values were calculated by a one-way ANOVA followed by Dunnett's multiple comparisons test ($* \, p$ <0.05, $** \, p$ <0.01, $*** \, p$ <0.01), compared to CT.

Supplemental information 2|

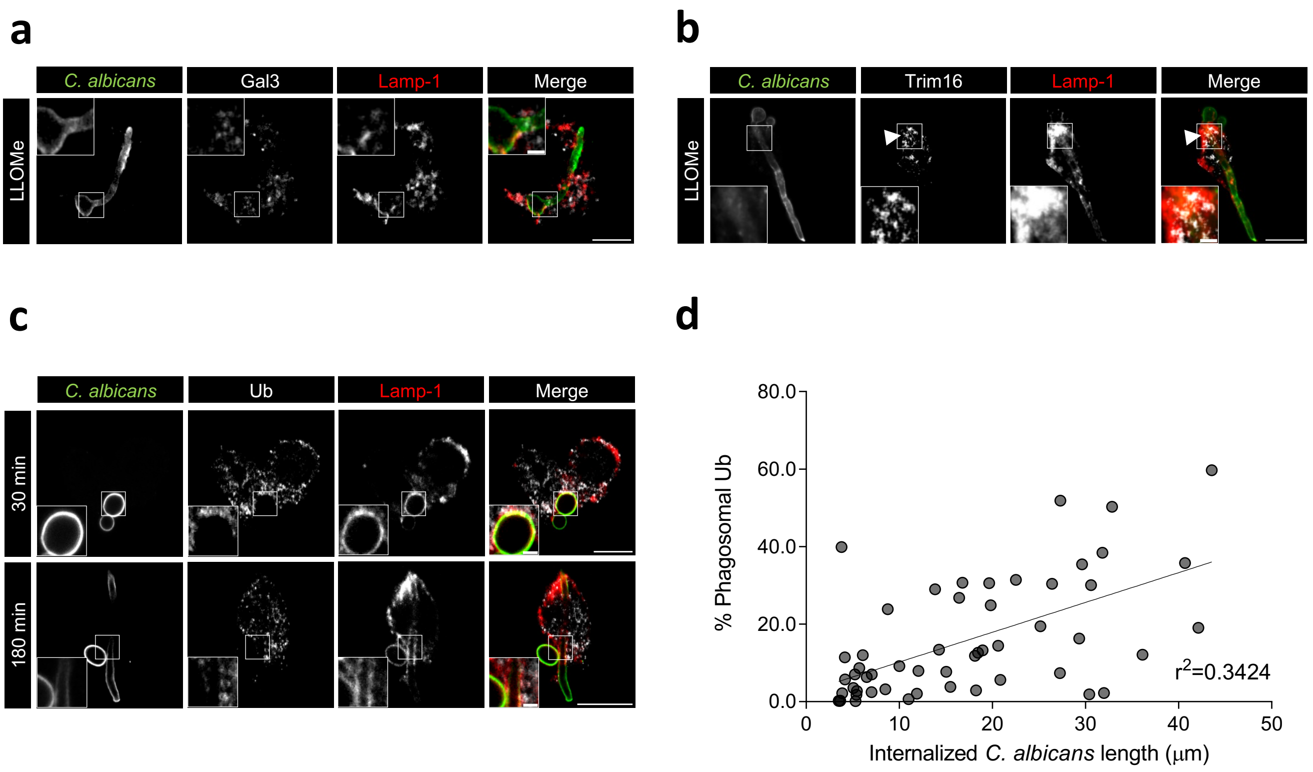

Fig S2 Recruitment of proteins involved in lysophagy process to the hyphae-containing phagolysosomes. (a-b) Representative confocal images of RAW cells infected with *C. albicans* and treated with LLOMe, fixed 180 min post-infection, and immunostained for *C. albicans*, Galectin-3 (Gal3) (a), Tripartite Motif Containing 16 (Trim16) (b) and Lamp-1. Scale bar, 10 µm and 2 µm. White arrows indicate sites of Trim16 accumulation. (c) Representative confocal images of RAW infected with *C. albicans* and treated with LLOMe, fixed 180 min post-infection, and immunostained for *C. albicans*, ubiquitin (Ub) and Lamp-1. Scale bar, 10 µm and 2 µm in the insets images. (d) Regression analysis comparing the length of internalized *C. albicans* and percent of phagosomal Ub quantified as (phagosomal Ub/total Ub)*100 per cell. Results represent the mean ± SEM from 3 independent experiments

Supplemental information 3|

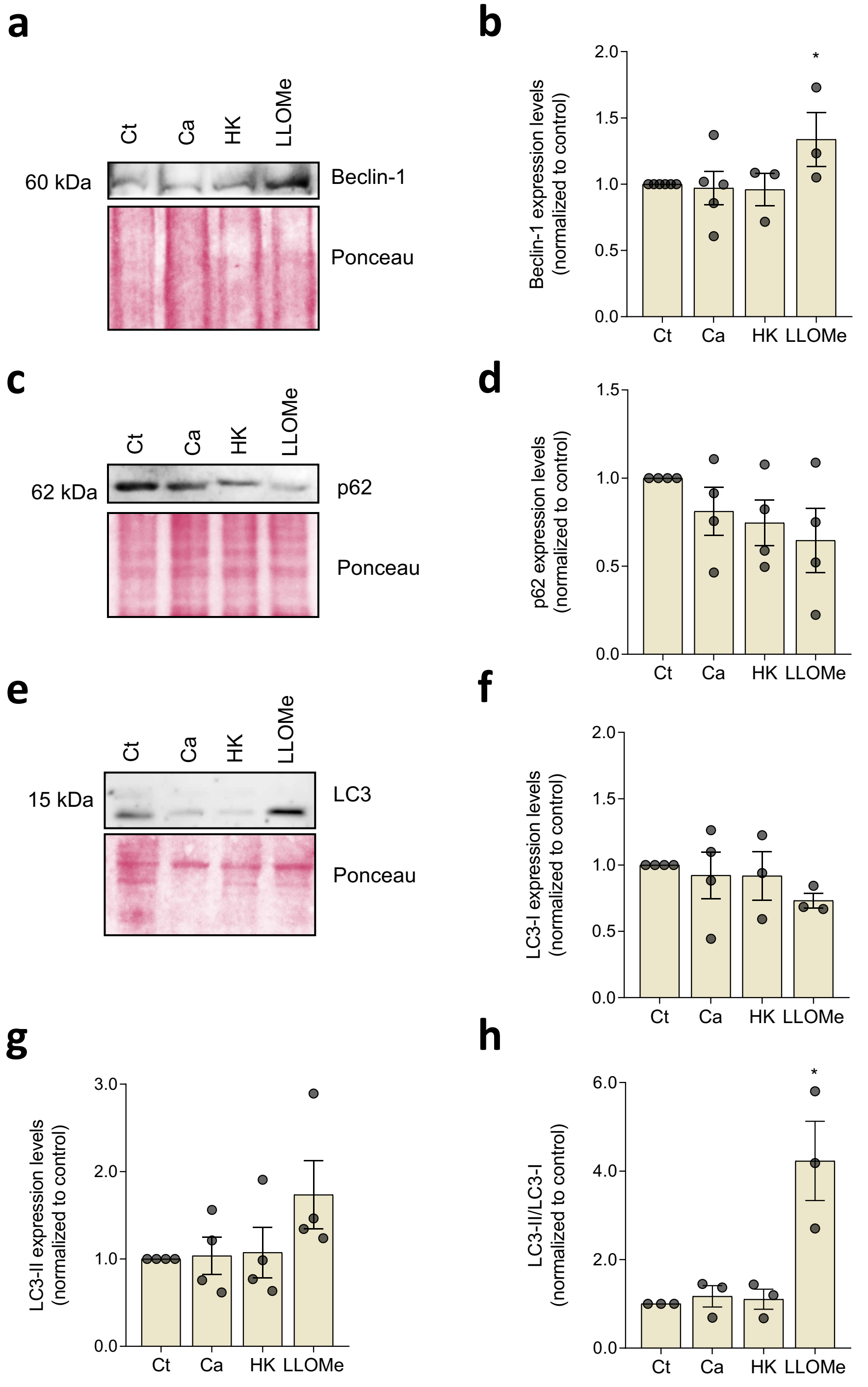

Fig S3 Macrophages infected with *C. albicans* did not present significant alterations in autophagy (a) Representative immunoblot image and (b) quantification of Beclin-1 protein total levels in RAW cells infected with *C. albicans* (*Ca*) or heat-killed (HK) *C. albicans*, or incubated with LLOMe, for 180 min. Results represent the mean ± SEM from 3 independent experiments. Ponceau staining was used as loading control. (c) Representative immunoblot image and (d) quantification of p62 protein levels in RAW cells infection with *Ca* or HK, or incubated with LLOMe, for 180 min. Results represent the mean ± SEM from 4 independent experiments. Ponceau staining was used as loading control. (e) Representative image and quantification (f-h) of immunoblot analysis of LC3-I, LC3-II and LC3-II/LC3-I protein levels in RAW cells infected with *Ca* or HK, or incubated with LLOMe, for 180 min. Results represent the mean ± SEM from 3 independent experiments. Ponceau staining was used as a loading control. p-values were calculated by a one-way ANOVA followed by Dunnett's multiple comparisons test ($*p$ < 0.05), compared to CT.

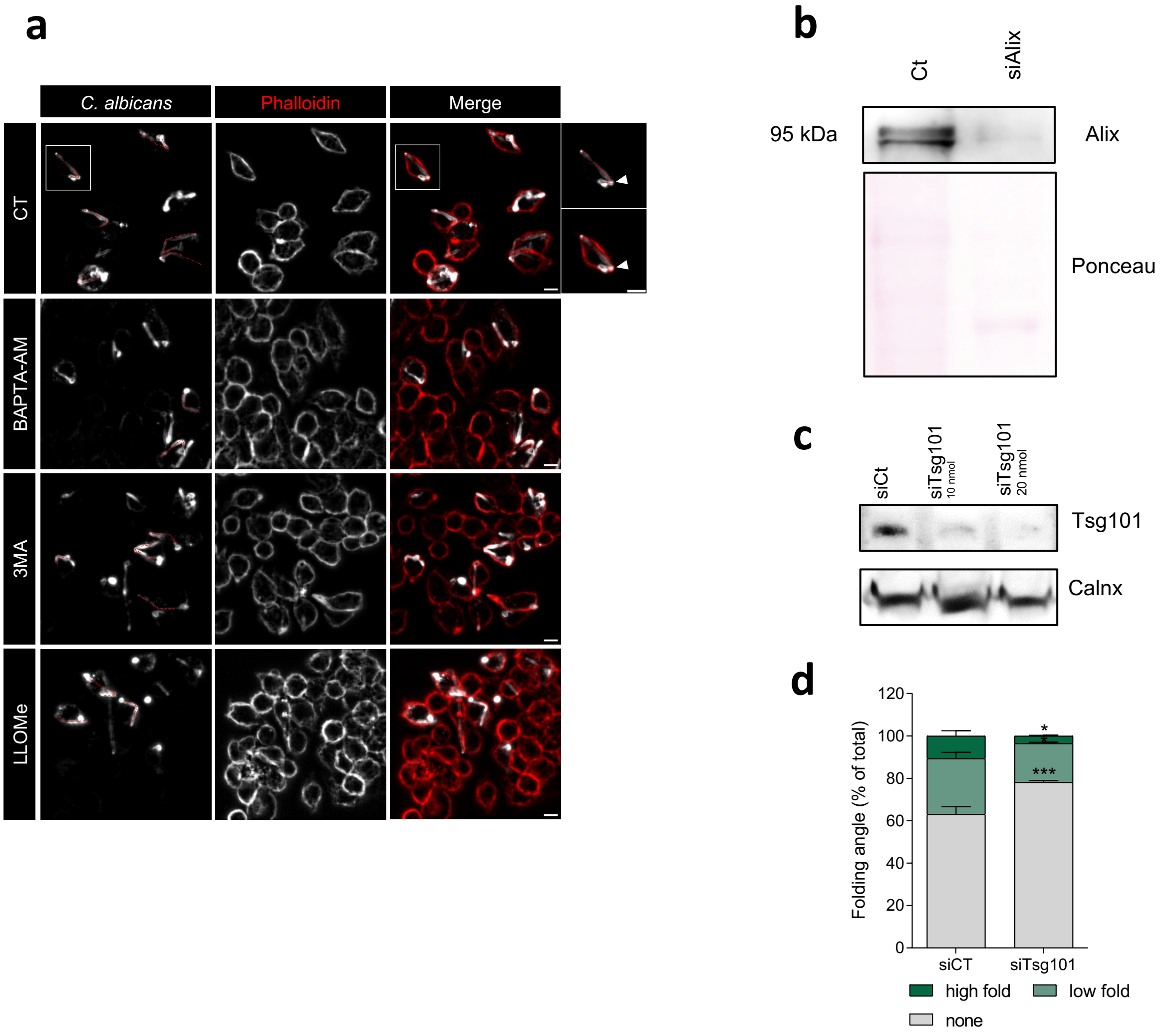

Fig S4 Representative images of RAW infected with *C. albicans* and treated with BAPTA-AM, 3MA and LLOMe, fixed 180 min post-infection, and immunostained for *C. albicans*, and Phalloidin. Scale bar, 10 µm and 2 µm in the insets. White arrows indicate sites of folding. Dotted line indicate folded *C. albicans.* (b-c) Alix and Tsg101 silencing was verified by immunoblotting, using Ponceau and calnexin (Calnx) as a loading control, respectively. (d) percentage of none, low and high angle of hyphae, upon infection of control and Tsg101-sillenced RAW cells with *C. albicans,* during 180 min. p-values were calculated by a t-test ($*p < 0.05$; $**p < 0.01$; $****p < 0.0001$).

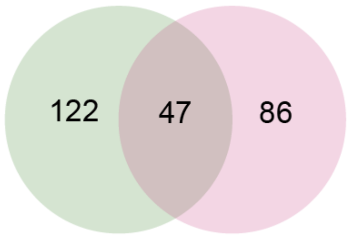

FIG S5 Venn diagram demonstrates the intersection of all the DEGs shared between vesicular trafficking and cell migration related GO term: cellular components.

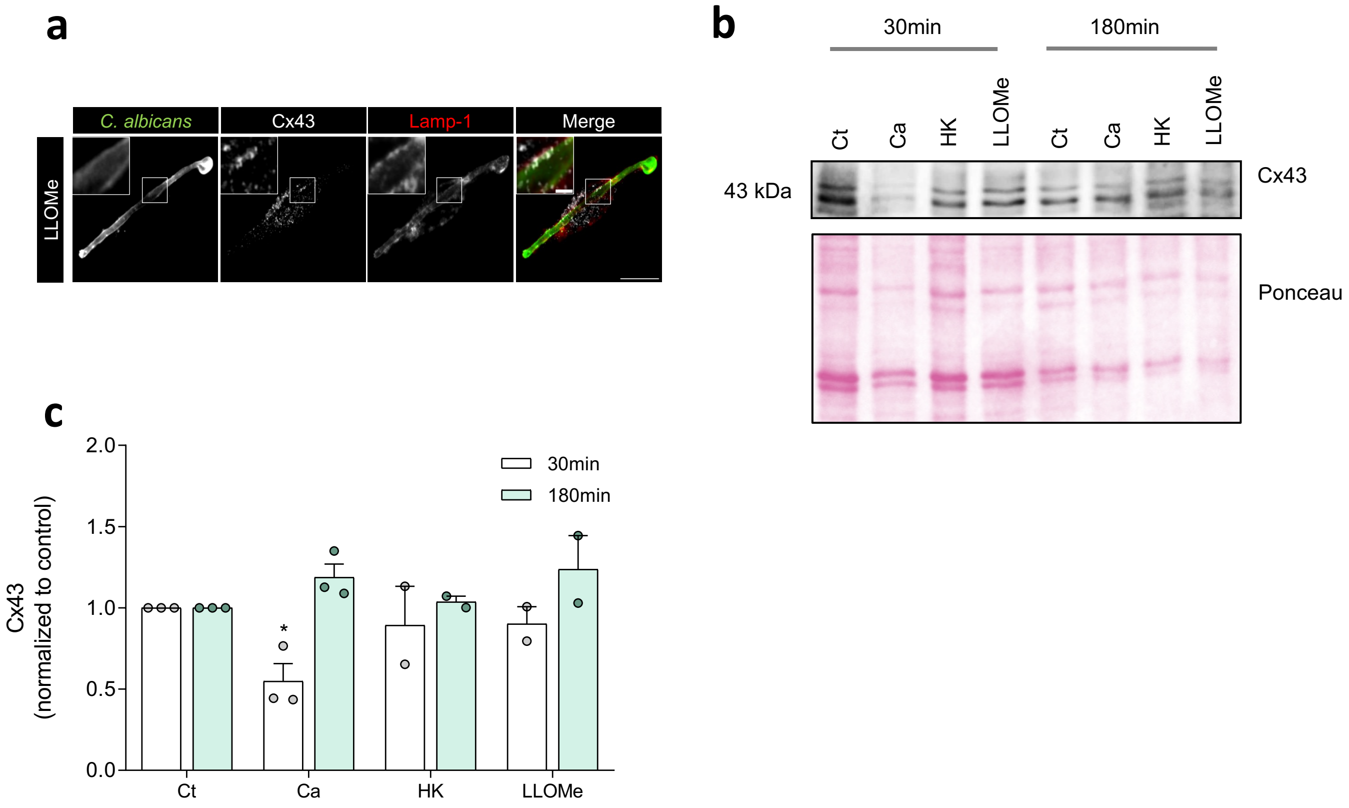

FIG S6 Cx43 distribution and levels during the macrophage infection with *C. albicans* (a) Representative confocal images of RAW infected with *C. albicans* and treated with LLOMe, fixed 180 min post-infection, and immunostained for *C. albicans*, Cx43 and Lamp-1. Scale bar, 10 µm and 2 µm in the inset images. (b) Representative immunoblot image and (c) quantification of Cx43 protein total levels in RAW cells infected with *C. albicans* (Ca) or heat-killed (HK) *C. albicans*, or incubated with LLOMe, for 30 or 180 min. Results represent the mean ± SEM from 3 independent experiments. Ponceau staining was used as loading control. p-values were calculated by a one-way ANOVA followed by Dunnett's multiple comparisons test ($*p$ < 0.05)

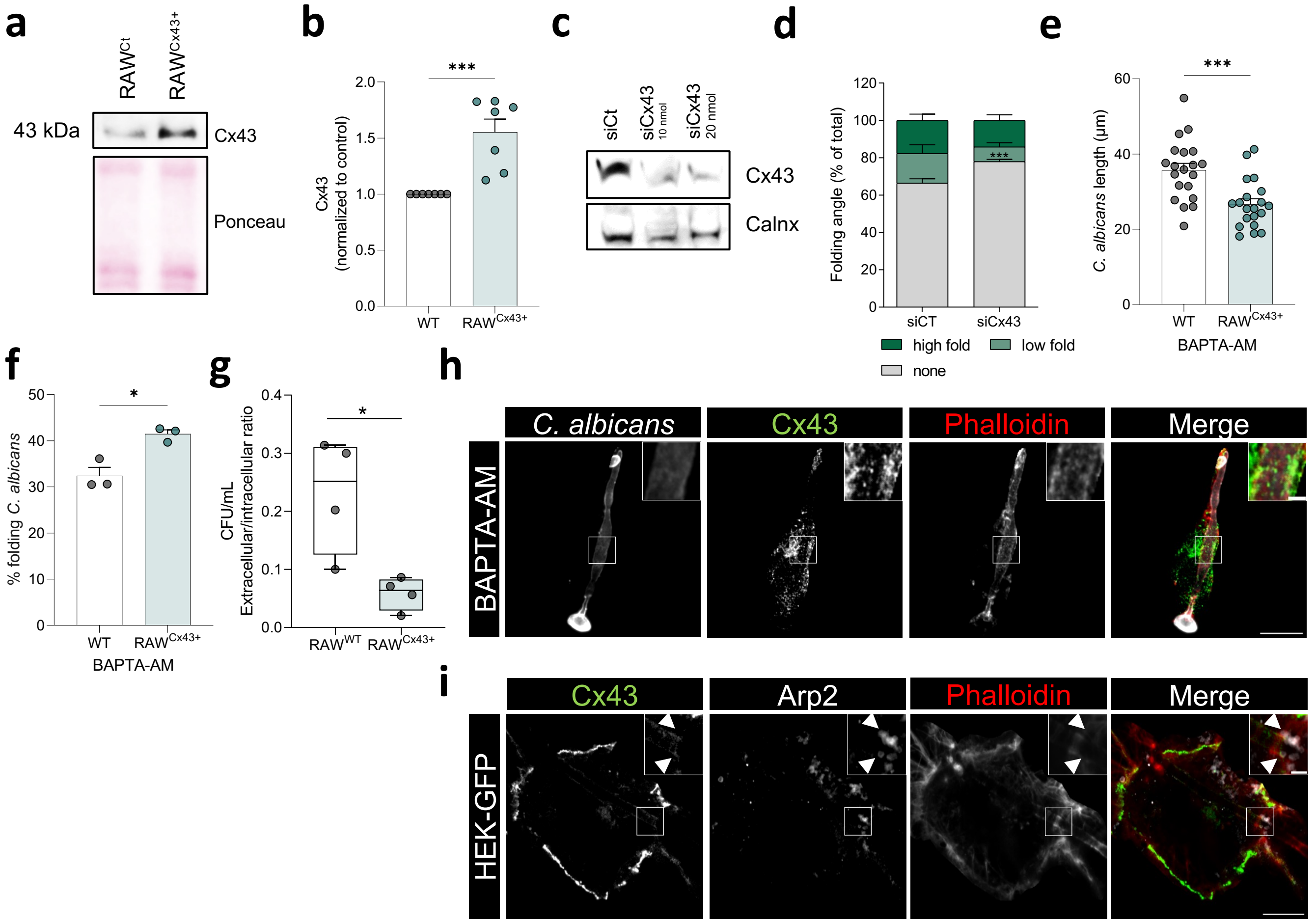

FIG S7 Increased Cx43 levels improve the cellular capacity to restrain internalized hyphae. (a-b) Cx43 overexpression was verified by immunoblotting, using Ponceau staining as a loading control. Results in (b) represent mean ± SEM from 7 independent experiments. (c) Cx43 depletion was verified by immunoblotting, using calnexin (Calnx) immunostaining as a loading control. (d) Quantification of *C. albicans* hyphal folding in control RAW cells (siCT) and Cx43-depleted cells (siCx43) for 180 min post-infection. Quantification of (e) *C. albicans* length and (f) percent of hyphal folding in control RAW (RAW^{WT}) and Cx43-overexpressing cells (RAW^{Cx43+}) and treated with BAPTA-AM, for 180 min post-infection. Results represent the mean ± SEM from 3 independent experiments. At least 10 cells were analysed per condition and independent experiment. p-values were calculated by a one-way ANOVA followed by Dunnett's multiple comparisons test ($*p < 0.05$; $***p < 0.001$), compared to Ct. (g) Ratio between the number of colony forming units (CFU) in the supernatant and in the cellular fraction (corresponding to intracellular fungae) at 3h post-infection. Data are means ± SEM (n = 4). (h) Representative confocal images of RAW infected with *C. albicans* and treated with BAPTA-AM, fixed 180 min post-infection, and immunostained for *C. albicans*, Cx43 and Phalloidin. Scale bar, 10 μm and 2 μm in the insets. (i) Representative confocal images of RAW cells infected with *C. albicans* during 180 min, and immunostained for Cx43, Arp2 and Phalloidin. Scale bar, 10 μm and 2 μm. White arrowheads indicate sites of Cx43 and Arp2 accumulation in the actin rings.
