# Supplementary material for: Cx43-mediated hyphal folding counteracts phagosome integrity loss during fungal infection
Source: Microbiol Spectr. 2023 Sep 21;11(5):e01238-23. doi: 10.1128/spectrum.01238-23 (PMC10581180; doi:10.1128/spectrum.01238-23)
Supplement: Supplemental Movie — Movie S1 [file spectrum.01238-23-s0002.pptx]

## Slide 1
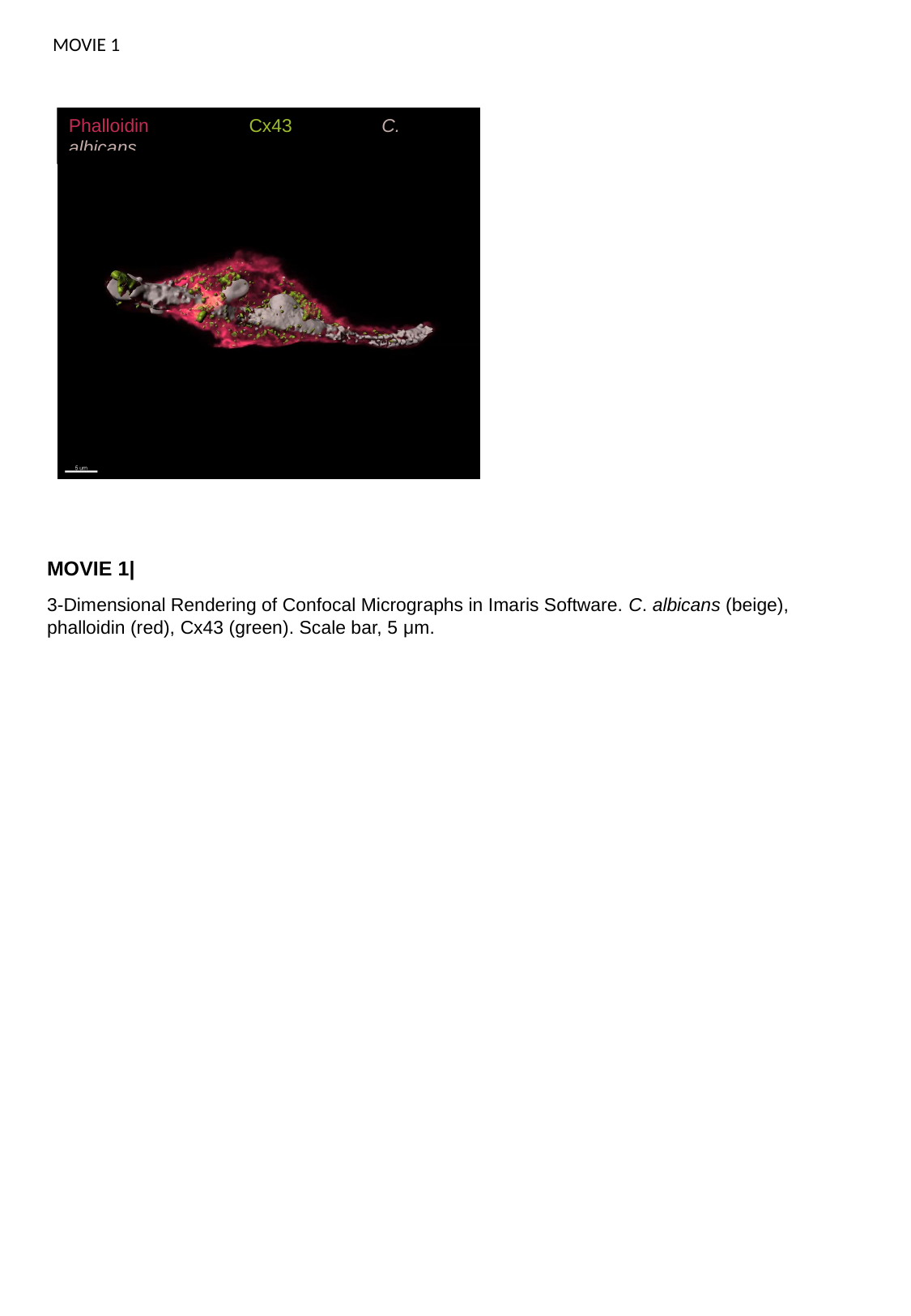

MOVIE 1
Phalloidin Cx43 C. albicans
MOVIE 1|
3-Dimensional Rendering of Confocal Micrographs in Imaris Software. C. albicans (beige), phalloidin (red), Cx43 (green). Scale bar, 5 μm.
